# Autopsy findings in cancer patients infected with SARS-CoV-2 show a milder presentation of COVID-19 compared to non-cancer patients

**DOI:** 10.1007/s11357-024-01163-7

**Published:** 2024-04-30

**Authors:** Éva Kocsmár, Ildikó Kocsmár, Flóra Elamin, Laura Pápai, Ákos Jakab, Tibor Várkonyi, Tibor Glasz, Gergely Rácz, Adrián Pesti, Krisztina Danics, András Kiss, Gergely Röst, Éva Belicza, Zsuzsa Schaff, Gábor Lotz

**Affiliations:** 1 Department of Pathology, Forensic and Insurance Medicine, Semmelweis University, Budapest, Hungary; 2 Department of Urology, Semmelweis University, Budapest, Hungary; 3 Department of Pathology and Experimental Cancer Research, Semmelweis University, Budapest, Hungary; 4 National Laboratory for Health Security, University of Szeged, Szeged, Hungary; 5 Hungarian Centre of Excellence for Molecular Medicine (HCEMM), Szeged, Hungary; 6 Health Services Management Training Centre, Faculty of Health and Public Administration, Semmelweis University, Budapest, Hungary

**Keywords:** SARS-CoV-2, COVID-19, Pneumonia, Cancer, Autopsy, Pathology

## Abstract

COVID-19, caused by SARS-CoV-2, manifests with differing severity across distinct patient subgroups, with outcomes influenced by underlying comorbidities such as cancer, which may cause functional and compositional alterations of the immune system during tumor progression. We aimed to investigate the association of SARS-CoV-2 infection and its complications with cancer in a large autopsy series and the role of COVID-19 in the fatal sequence leading to death. A total of 2641 adult autopsies were investigated, 539 of these were positive for SARS-CoV-2. Among the total number of patients analyzed, 829 had active cancer. Overall, the cohort included 100 patients who simultaneously had cancer and SARS-CoV-2 infection. The course of COVID-19 was less severe in cancer patients, including a significantly lower incidence of viral and bacterial pneumonia, occurring more frequently as a contributory disease or coexisting morbidity, or as SARS-CoV-2 positivity without viral disease. SARS-CoV-2 positivity was more frequent among non-metastatic than metastatic cancer cases, and in specific tumor types including hematologic malignancies. COVID-19 was more frequently found to be directly involved in the fatal sequence in patients undergoing active anticancer therapy, but less frequently in perioperative status, suggesting that the underlying malignancy and consequent surgery are more important factors leading to death perioperatively than viral disease. The course of COVID-19 in cancer patients was milder and balanced during the pandemic. This may be due to relative immunosuppressed status, and the fact that even early/mild viral infections can easily upset their condition, leading to death from their underlying cancer or its complications.

**Supplementary Information:**

The online version contains supplementary material available at 10.1007/s11357-024-01163-7.

## Introduction

The most formidable challenge for public health in recent decades has been COVID-19, an infectious disease instigated by the severe acute respiratory syndrome coronavirus (SARS-CoV-2). This pathogen has emerged as a significant threat, necessitating comprehensive investigation and understanding to address its profound implications on global health. Reported COVID-19 deaths in 2020–2021 totalled 5.94 million worldwide, but the full impact of the pandemic has been much greater. Excess mortality estimations suggest that 18.2 million people died worldwide over that period because of the COVID-19 pandemic [1]. In the five waves of the pandemic in Hungary until 2022 March, nearly 45,000 COVID-19-related deaths have been reported [2]. However, it is generally known that the virus can cause varying degrees of disease in different patient subgroups, depending on the comorbidities of the infected person [3]. Nevertheless, the published clinical studies do not provide a consistent picture of the factors influencing the severity of COVID-19. Several studies have demonstrated that both the infection itself and the severe outcome are more common in older people [4, 5]. In addition, a number of other comorbidities have been identified that negatively affect COVID-19 disease outcomes, with a prevalence that increases with age. Data have been reported that patients with cardiovascular diseases, diabetes, or hypertension have elevated ACE2 levels, combined with an increased risk of COVID-19 death [6].

Cancer patients are of particular interest from this point of view, since depending on the tumor character and the host’s immune environment, the cancer patients’ immune status can be functionally and compositionally altered during the tumor progression [7]. Some recent studies have found that cancer patients are more frequently affected by SARS-CoV-2 virus infection and have significantly higher mortality rates than non-cancer patients [4, 8, 9]. However, the role of cancer in influencing the progression of COVID-19 is complex. On the one hand, these patients are more frequently admitted to hospital for their underlying disease, resulting in more frequent COVID-19 testing, which also confirms asymptomatic infections [3, 10]. In addition, these patients spend significantly more time in hospital settings compared to the general population, and hospital environment is known to be a high-risk source of virus spread [11]. Radiotherapy and chemotherapy for cancer patients result in an immunosuppressed condition, which can also contribute to a more severe course of the disease [10]. Among cancer patients, COVID-19 patients with lung cancer and hematologic cancer show the worst prognosis [9, 12–14]. This is presumably due to poor respiratory function resulting from direct lung involvement in lung cancer patients and impaired humoral immunity in hematologic malignancies [13]. Nevertheless, the impact of distant metastases in advanced-stage cancers on COVID-19 outcomes remains controversial: while certain studies identify them as indicative of a poor outcome [14], others have not reported a significant association [4].

In addition, the controversial relationship between cancer and COVID-19 may be explained by understanding of immunometabolic pathways that intersect patients having infectious and neoplastic diseases simultaneously [3]. Macrophage activation plays a key role in the progression of both diseases. However, the cytokine storm seen in COVID disease is caused by M1-type macrophages, while anti-inflammatory M2-polarized macrophages are responsible for the anti-tumor immune response [3]. Furthermore, nicotinamide phosphoribosyltransferase (NAMPT) catalyses biosynthesis of nicotinamide adenine dinucleotide (NAD) from nicotinamide and NAMPT/NAD metabolism is altered in response to both viral infections and tumor growth, indirectly indicating the influence of tumor metabolic status on the progression of a patient’s COVID-19 disease [3]. Many aspects of the link between SARS-CoV-2 and cancer have been investigated, with results showing that the virus can influence the progression of the neoplastic disease on the level of glycolysis, translational modification, nucleic acid synthesis, lipid metabolism, and translational splicing [6].

Several clinical studies have investigated the incidence and outcome of SARS-CoV-2 infection in cancer patients. Our study is the first to analyze the association of SARS-CoV-2 infection and its complications with cancer in a large autopsy series, comparing the presentation of COVID-19 in cancer and non-cancer patients. We also aimed to investigate the role of COVID-19 in the fatal sequence leading to death and to analyze the influencing factors of the outcome of the disease in the light of additional clinicopathological parameters of the SARS-CoV-2-positive cancer patients.

## Methods

### Patient selection and definition of pandemic waves by temporal separation of them

Adult patients (> 18 years of age) who died between 04.03.2020 and 31.12.2022 at one of the clinics of Semmelweis University and underwent a complete pathological autopsy procedure at the Department of Pathology, Forensic and Insurance Medicine were included in the study.

The first and last day of an epidemic wave was defined as the day between two peaks of the epidemic curve, when the lowest daily case number was recorded. The starting date of the period under study is the date of the first domestic case, 4 March 2020. The epidemic curve was constructed using daily confirmed COVID-19 case numbers from the data collection of the National Public Health Centre (NPHC), with the date of confirmation of infection [2]. SARS-CoV-2-positive cases were analyzed only from the second wave onwards, as autopsies in the first wave were only allowed in Hungary under individual authorization. On this basis, patients were divided into four groups to compare the different waves: second wave (22.06.2020–24.01.2021), third wave (25.01.2021–04.07.2021), fourth wave (05.07.2021–26.12.2021), and 5 + waves according to the time between the start of the fifth wave and the end of the study period (27.12.2021–31.12.2022). In Hungary, the second wave was dominated by the wild-type SARS-CoV-2 variant, the third wave was caused by the Alpha (B.1.1.7) variant, the fourth wave by the Delta virus variant (B.1.617.2), while the 5 + waves were dominated by the Omicron BA.1 and BA.2 variants [15, 16].

### Ethical approval

The study protocol was following the ethical guidelines of the 1975 Declaration of Helsinki and was approved by the Central Ethical Medical Committee, Budapest (IV/3961–2/2020/EKU, 4354–1/2022/EKU and IV/1543—1/2022/EKU).

### Autopsy procedure and reporting

In all cases, the autopsies and the following histological examinations of the patients were performed by a board-certified pathologist or by a pathology resident under the professional supervision of a board-certified pathologist. Tissue sampling for postmortem histopathological examination was done from at least nine different localizations, determined by the questions raised during the autopsy but typically included the heart, lungs, liver, kidneys. Moreover, a much more extended sampling was done according to a predefined protocol during autopsy of SARS-CoV-2-positive cases, including (but not limited to) the following organs/localizations: (1) heart—left ventricle, (2) heart—right ventricle, (3) trachea, (4–5) right lung—upper lobe, (6) right lung—middle lobe, (7–8) right lung—lower lobe, (9–10) left lung—upper lobe, (11–12) left lung—lower lobe, (13) spleen, (14) pancreas—head, (15) liver—right lobe, (16) liver—left lobe, (17) thyroid gland—right lobe, (18) kidney—right, (19) adrenal gland—right, (20) sinus cavity, (21) oropharynx.

When determining the diagnoses of each autopsy case, the different diseases (clinically and/or autopsy diagnosed) were categorized according to their role in the fatal sequence leading to death or their independence from it. The categorization widely used in autopsy pathology describes the events leading to death as a chain of three components (“fatal sequence”): the underlying disease which directly initiates the process leading to death, its complications which further drive this process, and the final of these which is the direct/immediate cause of death. The contributory disease is not included in the preceding sequence (underlying disease-complications-immediate cause of death), but worsens the consequences of the underlying disease and its complications. Another type of diagnosis is concomitant findings not related to the chain of fatal events (coexisting morbidity), which means that they are completely unrelated diseases that do not contribute in any way to the fatal sequence.

When investigating the association of patients’ deaths with COVID-19 disease or cancer, the cases were divided into three groups according to the involvement of SARS-CoV-2 infection or malignant disease. In the first group, the investigated entity (SARS-CoV-2 infection/cancer) was part of the fatal sequence leading to death (underlying cause of death, its complications as consequential diseases, and immediate/direct cause of death), while the second group included cases in which SARS-CoV-2 infection or cancer was a contributory disease and in the third group the investigated entity was only a concomitant finding not related to the chain of fatal events (coexisting morbidity). The diagnosis of a COVID-19-related lung disease was based on macroscopic and/or histological examination of the lungs. Diagnosis of bacterial pneumonia was based on the macroscopic appearance of purulent inflammation in the lung parenchyma (presence of pus). Subsequently, a histological examination was also performed, during which the presence of purulent pneumonia in the lung tissue was confirmed histopathologically (by identification of filling of the alveoli of the lung with neutrophil granulocytes).

The diagnosis list of each autopsy report has been supervised and standardized by experts for the study (ÉK, GL).

Patients were considered to be cancer patients if they had a clinical history of cancer that required medical treatment or affected their condition within a year before their admission to hospital or if the autopsy procedure confirmed cancer. Patients who had a history of cancer but whose disease was eliminated by treatment (more than 1 year) and whose previous cancer did not affect their current condition were categorized as non-cancer patients in the study.

### Clinical data and antemortem SARS-CoV-2 testing of the autopsied patients

The clinical data of the patients were collected from the electronic registry of Semmelweis University.

Full vaccination status was attributed to patients who had completed the primary series of any of the vaccines authorized in Hungary throughout the study duration. The primary series typically comprised two doses for the majority of vaccines; those who received fewer than this were classified as partially vaccinated. Patients with no information on their vaccination history in the university’s electronic system were considered to have unknown vaccination status.

An antemortem clinical sampling was carried out from the upper respiratory tract of each patient for SARS-CoV-2 testing in accordance with the patient care regulations of Semmelweis University, Budapest, and the Interim Guidelines for Collecting and Handling of Clinical Specimens for COVID-19 Testing (Centers for Disease Control and Prevention of the United States of America; https://www.cdc.gov/coronavirus/2019-ncov/lab/guidelines-clinical-specimens.html).

Patients were considered SARS-CoV-2-positive if they had a positive antemortem real-time reverse-transcription polymerase chain reaction test (AzureSeq-200 CE RT-qPCR Kit SARS-CoV-2, Omixon Biocomputing Ltd., Budapest, Hungary) or antigen rapid test (Panbio™ COVID-19 Antigen Self-Test, Abbott Rapid Diagnostics Jena GmbH, Jena, Germany) during their hospital admission or stay and were still a positive patient at the time of death.

### Statistical analysis

All data management, calculations, and plotting were carried out in R software environment (version 4.0.2) and R Studio portable (version 1.3.959).

The following parameters were investigated between subgroups of patients stratified by SARS-CoV-2 positivity and cancer: sex (male/female), age (in years), vaccination status (vaccinated/unvaccinated/partly vaccinated/unknown), complications of SARS-CoV-2 infection (viral pneumonia/viral pneumonia with bacterial overinfection/asymptomatic infection), role of COVID-19 in death (COVID-19 in sequence leading to death/contributory disease/concomitant disease), role of cancer in death (cancer in sequence leading to death/contributory disease/concomitant disease), COVID-19 waves (2nd/3rd/4th/the time between the start of fifth wave and the end of the study), primary tumor type (breast/colorectal/gastroesophageal/genitourinary/gynecologic/head and neck/hematologic/liver/lung/pancreatobiliary/multiple/other), and presence of distant metastasis (yes/no). Categorical variables were described as frequencies and analyzed using 2 × 2 or 2 × 3 contingency tables and compared using Fisher’s exact probability test and paired Wilcoxon test. Continuous variables were described as means, range, and SD and age differences were analyzed by the Kruskal–Wallis test. Univariable and multivariable logistic regression analyses were used to identify the independent risk factors for having SARS-CoV-2 infection among conditions of the fatal sequence leading to death. Using the MASS package in the R software environment, multivariable analysis with backward selection method was also performed to select variables associated with the fatal role of COVID-19 or the type of the developed pneumonia. The parameters examined were the following: age (years), sex (male/female), vaccination (yes/partly (at least one shot)/no or not known), anti-COVID-19 treatment (no/yes), perioperative status (within 30 days after surgery; no/yes), hospitalization (days), primary tumor (solitary/multiple or metastatic or hematologic), under ongoing anticancer therapy (no/yes). All *p* values were calculated two-tailed and considered significant when *p* < 0.05.

### Population-based mortality data of cancer patients infected with SARS-CoV-2

To investigate the impact of SARS-CoV-2 infection on mortality among Hungarian cancer patients, a retrospective case–control analysis was also conducted. In this part of the study, we focused on mortality from two common malignant solid tumor types, so that patients with ICD-10 codes of colorectal cancer (CRC) (C17-C20) or breast cancer (C50) were included in the investigated cases and the population without a diagnosis of C17-C20 or C50 was included in the control cases. The analysis method was logistic regression using IBM SPSS 27.0 software. Patients eligible for the study were identified from anonymized administrative medical data of the National Health Insurance Fund Management (NEAK) using the social security identification number (TAJ) database. The outcome variable was mortality after 1 January 2020; independent variables were age, sex (for CRC), medical history of comorbidities, number of SARS-CoV-2 infections, number of COVID-19 vaccinations, and C50 or CRC ICD status. The disease-specific codes were defined by an oncologist. Inclusion criteria for the study were as follows: Hungarian citizen, valid Hungarian social security identification number (TAJ), registered at least once by the National Center for Public Health and Pharmacy (NNGYK) as infected with SARS-CoV-2, alive on January 1, 2020, and at least 40 years old. Databases used were as follows: inpatient, outpatient, and TAJ (containing data on residents’ demographics, citizenship, TAJ validity) databases of the NEAK from the period of 2010–2021; e-Health Care Cloud Hosting (EESZT) vaccination data registry; NNGYK SARS-CoV-2 infection registry. More details are shown in the Supplementary material.

## Results

### Cohort characteristics

A total of 2641 adult autopsies were performed in our department during the study period. A total of 539 of these patients tested positive for SARS-CoV-2 on antemortem testing. Among the total number of patients analyzed, 829 had active cancer. In terms of primary tumors, lung cancer was the most common (123 cases, 14.8%), followed by cancers of genitourinary origin (116 cases, 14%), colorectal cancer (112 cases, 13.5%), hematologic malignancies (102 cases, 12. 3%), pancreatobiliary cancers (94 cases, 11.3%), head and neck cancers (46 cases, 5.6%), breast cancer (44 cases, 5.3%), stomach or oesophageal cancer (38 cases, 4.6%), liver cancer (26 cases, 3.1%), and cancer with gynaecological origin (24 cases, 2.9%). A total of 40 patients (4.8%) in the study cohort had more than one tumor diagnosis simultaneously. A further 63 patients (7.6%) had less common tumors, distributed as follows: cancer of unknown primary origin (CUP) (15 cases), central nervous system tumor (13 cases), soft tissue tumor (9 cases), melanoma (8 cases), endocrine tumor (7 cases), skin cancer (3 cases), mesothelioma (4 cases), small bowel cancer (3 cases) and appendix tumor (1 case). Overall, the cohort included 100 patients who simultaneously had cancer and SARS-CoV-2 infection.

### Clinicopathological parameters of SARS-CoV-2-infected cancer and non-cancer patients

The characteristics of SARS-CoV-2-infected cancer and non-cancer patients autopsied in our department during the COVID-19 pandemic are shown in Table 1. No statistically significant difference was found in the distribution of the age, sex, and vaccination status. However, significant differences were found in the presentation of the infection, the role of COVID-19 in death, and the distribution of the cancer vs non-cancer patients by COVID-19 waves. The presentation of the infection in the SARS-CoV-2-infected cancer patients was milder as both the viral and bacterial pneumonia were significantly less frequent (42.0% and 24.0%, respectively) in comparison with the non-cancer cases (52.6% and 32.8%, respectively) while a higher proportion of cancer patients had SARS-CoV-2 infection without COVID-19 disease (34.0% vs. 14.6% in non-cancer patients). Another important difference is that COVID-19 more frequently played a direct role in the death of the non-cancer patients (75.6% in non-cancer vs. 45.0% in cancer patients) while it was more frequently found as contributory disease or coexisting morbidity in the cancer patients (10.3% and 14.1% in non-cancer vs. 22.0% and 33.0% in cancer cases, respectively). The distribution of autopsied non-cancer cases showed a relatively balanced distribution in the different waves of COVID-19, while a higher proportion of cancer cases among the deceased infected patients was observed in the second wave.

**Table 1:** Comparison of baseline clinicopathological parameters and characteristics of SARS-CoV-2 infection between SARS-CoV-2-positive cancer and non-cancer patients

Parameter		Total	Cancer	Non-cancer	*p*
Number of patients	*n*	539	100	439	-
Age	Mean, range	69.54 (23–101)	70.31 (27–91)	69.36 (23–101)	0.8599
Sex	Female (*n*)	223 (41.4%)	35 (35.0%)	188 (42.8%)	0.1771
	Male (*n*)	316 (58.6%)	65 (65.0%)	251 (57.2%)	
Vaccination status	Vaccinated	130 (24.1%)	30 (30%)	100 (22.8%)	0.1448
	Partly	36 (6.7%)	3 (3.0%)	33 (7.5%)	
	Unvaccinated	203 (37.7%)	42 (42.0%)	161 (36.7%)	
	Unknown*	170 (31.5%)	25 (25.0%)	145 (33.0%)	
Complications of COVID-19	Viral pneumonia	273 (50.6%)	42 (42.0%)	231 (52.6%)	** < 0.0001**
	Bacterial pneumonia	168 (31.2%)	24 (24.0%)	144 (32.8%)	
	Only infection	98 (18.2%)	34 (34.0%)	64 (14.6%)	
Role of COVID-19 in death	Direct cause of death	377 (70.0%)	45 (45.0%)	332 (75.6%)	** < 0.0001**
	Contributory disease	67 (12.4%)	22 (22.0%)	45 (10.3%)	
	Coexisting morbidity	95 (17.6%)	33 (33.0%)	62 (14.1%)	
COVID-19 waves	2nd	160 (29.7%)	40 (40.0%)	120 (27.3%)	**0.0003**
	3rd	158 (29.3%)	17 (17.0%)	141 (32.1%)	
	4th	97 (18.0%)	11 (11.0%)	86 (19.6%)	
	5th	124 (23.0%)	32 (32.0%)	92 (21.0%)	

^*^Patients with unknown vaccination status were excluded from this comparison. The result *p* = 0.1448 is obtained from a 3 × 2 comparison of vaccinated, partially vaccinated and unvaccinated subgroups

### Characteristics of cancer patients with or without SARS-CoV-2 infection

The characteristics of SARS-CoV-2-infected and non-infected cancer patients who underwent autopsy in our department during the COVID-19 pandemic are shown in Table 2. No statistically significant difference was found in the distribution of the age and sex between the virus-infected and SARS-CoV-2-negative subgroups. However, in SARS-CoV-2-negative cancer patients, malignant disease was significantly more often the direct cause of death (76.3% in SARS-CoV-2-negative vs 60.0% in infected patients) and less frequently a contributory disease or coexisting morbidity (18.2% and 5.5% in uninfected vs 36.0% and 4.0% in infected patients). In line with this, distant metastases were more frequently seen in the SARS-CoV-2-negative cancer patients. The distribution of cases according to primary tumor type showed a relatively higher occurrence of hematologic malignancies among SARS-CoV-2-positive cancer patients compared with the uninfected subgroup, in which lung cancer was slightly more common.

**Table 2:** Comparison of basic clinicopathological parameters and tumor characteristics between SARS-CoV-2-positive and -negative cancer patients

Parameter		Total	SARS-CoV-2 positive	SARS-CoV-2 negative	*p*
Number of patients	*n*	829	100	729	-
Age	Mean, range	71.06 (20–97)	70.31 (27–91)	71.16 (20–97)	0.4877
Sex	Female (*n*)	352 (42.5%)	35 (35.0%)	317 (43.48%)	0.1307
	Male (*n*)	477 (57.5%)	65 (65.0%)	412 (56.52%)	
Role of CANCER in death	Direct cause of death	616 (74.3%)	60 (60.0%)	556 (76.27%)	**0.0004**
	Contributory disease	169 (20.4%)	36 (36.0%)	133 (18.24%)	
	Coexisting morbidity	44 (5.3%)	4 (4.0%)	40 (5.49%)	
Primary tumor	Breast	44 (5.3%)	4 (4.0%)	40 (5.49%)	**0.0025**
	CRC	112 (13.5%)	10 (10.0%)	102 (13.99%)	
	Gastroesophageal	38 (4.6%)	5 (5.0%)	33 (4.53%)	
	Genitourinary	116 (14.0%)	13 (13.0%)	103 (14.13%)	
	Gynecologic	24 (2.9%)	2 (2.0%)	22 (3.02%)	
	Head and neck	46 (5.6%)	2 (2.0%)	44 (6.04%)	
	Hematologic	102 (12.3%)	26 (26.0%)	76 (10.43%)	
	Liver	26 (3.1%)	4 (4.0%)	22 (3.02%)	
	Lung	123 (14.8%)	10 (10.0%)	113 (15.50%)	
	Pancreatobiliary	94 (11.3%)	9 (9.0%)	85 (11.66%)	
	Multiple	40 (4.8%)	6 (6.0%)	34 (4.66%)	
	Other	63 (7,6%)	8 (8.0%)	55 (7.55%)	
Distant metastasis*	yes	410 (49.5%)	28 (28.0%)	382 (52.4%)	**0.0008**
	No	317 (38.2%)	46 (46.0%)	271 (37.2%)	

*CRC* colorectal cancer

^*^Cases with hematologic malignancies were excluded from this analysis

**Fig. 1:**
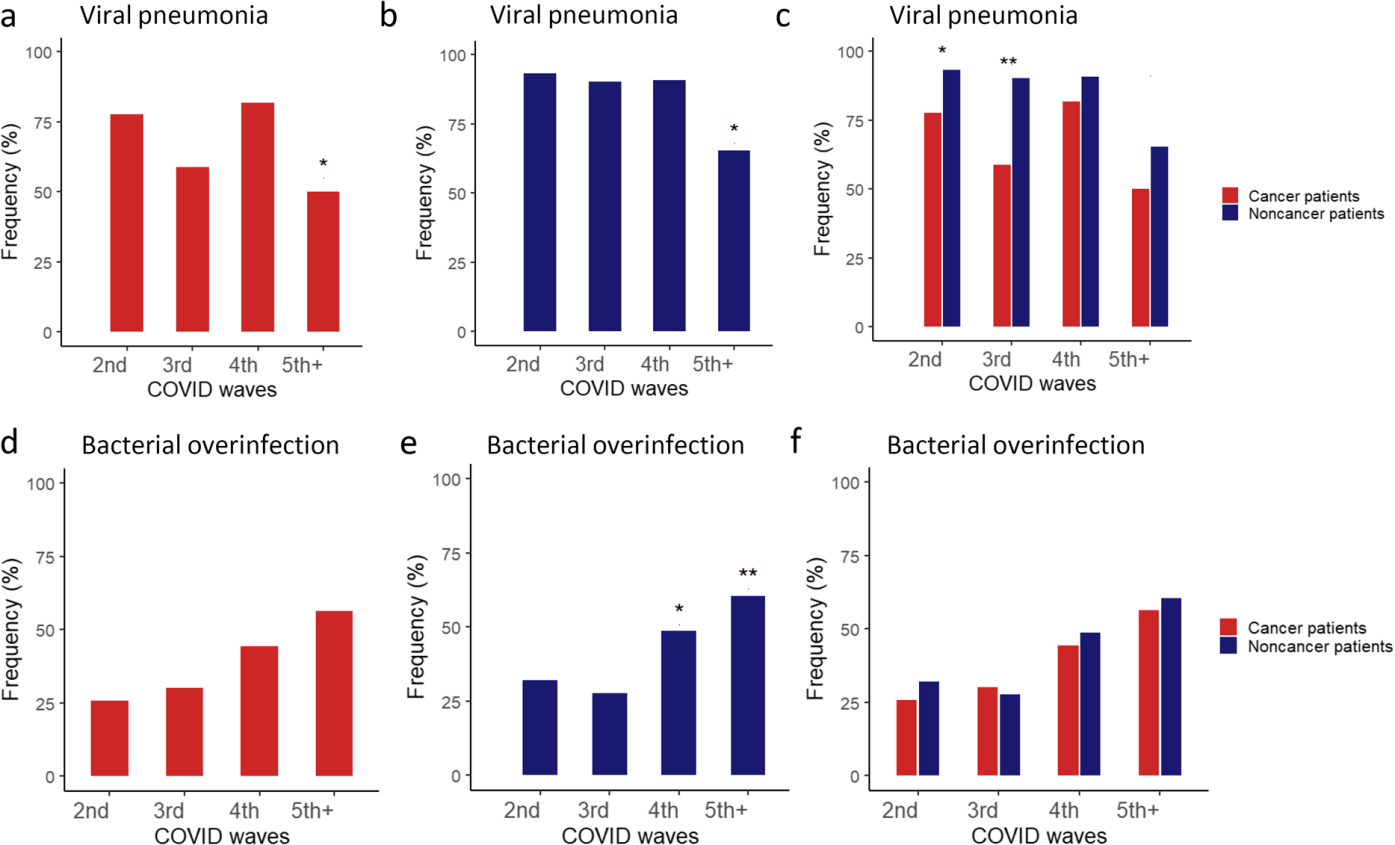
Presence of viral pneumonia (a, b, c) and its bacterial overinfection (d, e, f) in SARS-CoV-2 infected cancer (red columns) and non-cancer (blue columns) patients

Cancer and non-cancer patients were investigated to determine whether the infection had resulted in viral pneumonia (with or without bacterial overinfection). Our results indicated that both cancer and non-cancer groups of patients were significantly less likely to develop pneumonia during the fifth wave, as compared to the second wave (Fig. 1a, b; *p* = 0.0243 for cancer and *p* < 0.0001 for non-cancer patients). Furthermore, when comparing the two groups of patients in the different waves, the occurence of pneumonia was significantly more frequent in the second (*p* = 0.0139) and third (*p* = 0.0022) waves among non-cancer patients, but not in waves 4 and 5 + (Fig. 1c).

Among pneumonia cases, the presence of bacterial overinfection showed a similar increasing trend towards later waves between cancer and non-cancer patients, but no statistically significant difference between waves was found in cancer patients (Fig. 1d), whereas among non-cancer patients, the rate of bacterial complications was significantly higher in the waves 4 (*p* = 0.0239) and 5 + (*p* = 0.0005), compared to the wave 2 (Fig. 1e). When the two groups of patients were compared, no significant differences were found in the different waves, confirming the similarity of the increasing trend in the incidence of bacterial overinfection in the later waves between cancer and non-cancer patients (Fig. 1f).

**Fig. 2:**
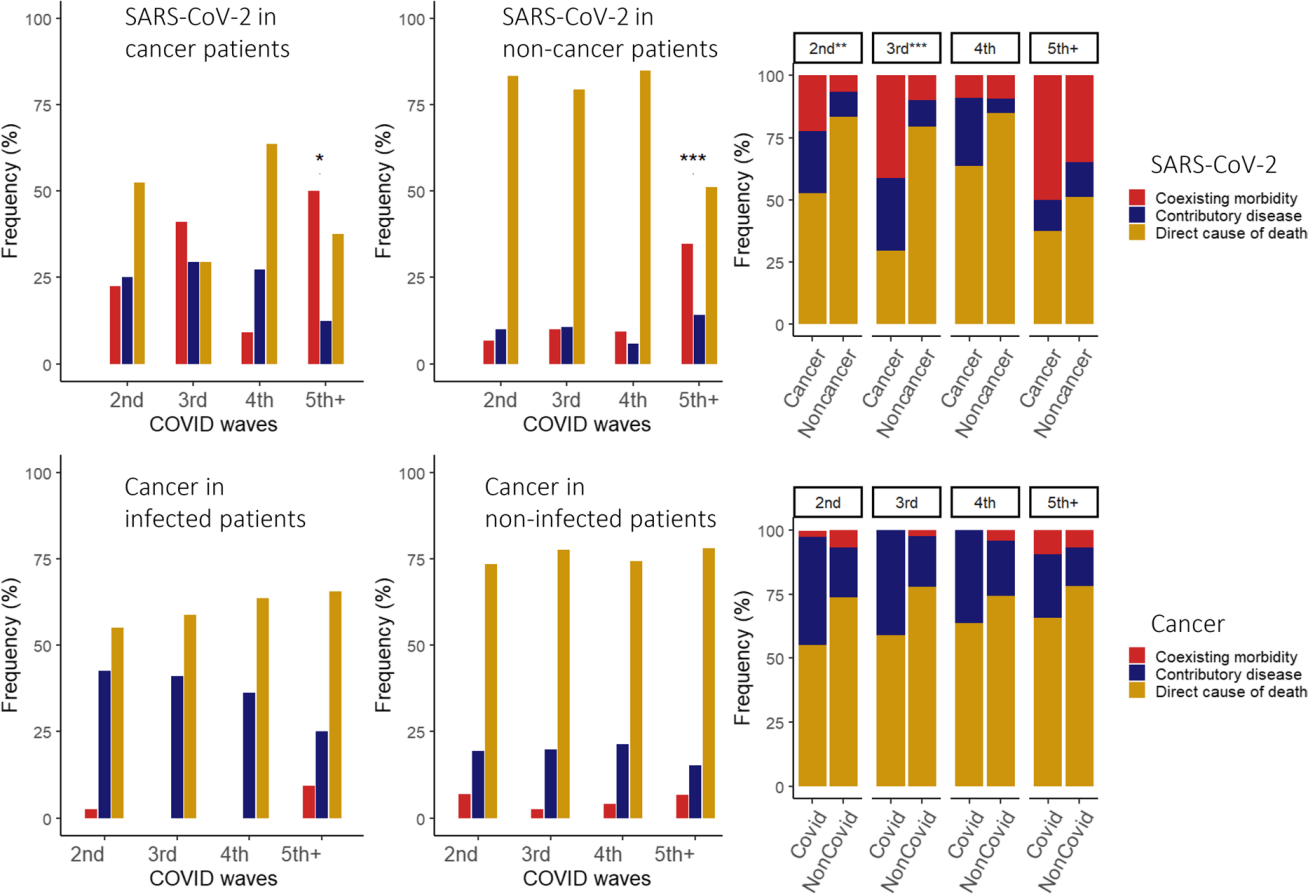
Role of SARS-CoV-2 infection and cancer in the fatal sequence. Contribution of SARS-CoV-2 infection (a: in cancer, b: in non-cancer, c: in both cancer and non-cancer patients) and cancer (d: in SARS-CoV-2 infected, e: in non-infected, f: in both SARS-CoV-2 infected and non-infected patients) to the fatal sequence in the 2nd, 3rd, 4th and 5 + waves

We also investigated the role of SARS-CoV-2 infection and cancer in the sequence leading to death. COVID-19 was significantly more common concomitant disease/coexisting morbidity (not related to fatal sequence) in the waves 5 + compared to the second wave in both cancer (*p* = 0.0497) and non-cancer (*p* < 0.0001) patients (Fig. 2a, b). However, when comparing these two groups of patients in the different waves, SARS-CoV-2 infection/COVID-19 diagnosis as coexisting morbidity (not related to fatal sequence) was more frequent in the second (*p* = 0.0004) and third (*p* < 0.0001) waves among the cancer than in non-cancer patients (Fig. 2c). Conversely, when we analyzed the role of cancer in the chain of events leading to death in the SARS-CoV-2 infected and uninfected subgroups, we found no significant difference between either wave compared to the second wave, and no significant difference between the two subgroups in the different waves (Fig. 2d–f).

*Contribution of COVID-19 to death in SARS-CoV-2-infected cancer patients.*

We performed univariable and multivariable logistic regression to identify the clinical factors that might be associated with the direct role of SARS-CoV-2 infection in the fatal sequence. In univariable analysis, gender, specific anti-COVID-19 therapy (any of remdesivir, favipiravir, REGEN-COV), ongoing (active) chemotherapy, perioperative status (within 30 days after surgery), and tumor dissemination status (solitary primary/multiple primary/metastatic/hematologic) were the factors that significantly influenced whether COVID-19 played a direct role in the sequence leading to death (Table 3a). Multivariable logistic regression analysis revealed that perioperative status is a negative independent predictor of the direct involvement of COVID-19 disease in the fatal sequence (*p* = 0.0123; OR = 0.17, CI = 0.04–0.60). In contrast, among patients who were currently undergoing anti-tumor therapy, COVID-19 was independently positively associated with direct involvement of the disease in the chain of events leading to death (*p* = 0.0167, OR = 4.76, CI = 1.43–19.38) (Table 3a). However, when looking for which clinical factors have a significant influence on whether COVID-19 plays any role (not only a direct but also a contributing role) in cancer mortality, the association with active anticancer treatment was found to be low, as nor univariable, nor multivariable logistic regression analysis did not show a significant association, while a multivariable logistic regression with backward selection approach found a marginally significant *p*-value (0.044). Moreover, sex, pandemic waves, and perioperative status were found to be significant influencing factors in univariable analysis, out of which pandemic waves were also found to be a significant independent influencing factor of the underlying role of COVID-19 in the sequence leading to death in multivariable analysis (Table 3b). The pandemic waves also appeared to be a significant independent determinant of the clinical presentation of COVID-19 disease in SARS-CoV-2-infected cancer patients, as the incidence of pneumonia differed significantly between waves (Fig. 1a and c, Table 3c).

**Table 3:** Clinical factors influencing the contribution of COVID-19 to death and the development of pneumonia in SARS-CoV-2-infected cancer patients

a) COVID-19 directly involved in the fatal sequence vs. COVID-19 as contributory or coexisting disease		Univariable		Multivariable I		Multivariable II (backward selection)	
		OR (95% CI)	*p*	OR (95% CI)	*p*	OR (95% CI)	*p*
Age	Years	0.99 (0.95–1.03)	0.610	0.998 (0.96–1.04)	0.9220		
Sex	Male vs. female	0.42 (0.17–0.97)	0.0477	0.478 (0.17– 1.32)	0.1531	0.407 (0.15–1.02)	0.0605
Waves	2–3-4 vs. 5 +	0.64 (0.26–1.49)	0.303	0.372 (0.096–1.44)	0.1512		
Vaccination	Yes/partly vs. no/not known	1.23 (0.53–2.86)	0.623	1.714 (0.46–6.37)	0.4211		
Anti-COVID-19 treatment	No vs. yes	3.10 (1.24–8.19)	0.0176	2.218 (0.78–6.34)	0.1369		
Perioperative status	No vs. yes	0.36 (0.13–0.99)	0.0088	0.192 (0.05–0.80)	**0.0233**	0.17 (0.04–0.60)	**0.0123**
Hospitalization	Days	0.99 (0.97–1.02)	0.614	0.989 (0.96–1.02)	0.5349		
Primary tumor	Solitary vs. disseminated (multiple/metastatic/hematologic)	1.51 (0.68–3.43)	0.318	0.907 (0.35–2.39)	0.8439		
Under chemotherapy	No vs. yes	4.09 (1.05–27.18)	0.0132	4.799 (1.19- 19.4)	**0.0278**	4.76 (1.43–19.38)	**0.0167**
b) COVID-19 contributing to death in any way (directly or as contributor) vs. COVID-19 as coexisting morbidity		Univariable		Multivariable I		Multivariable II (backward selection)	
		OR (95% CI)	*p*	OR (95% CI)	*p*	OR (95% CI)	*p*
Age	Years	1.01 (0.97–1.05)	0.656	1.024 (0.98–1.07)	0.3274		
Sex	Male vs. female	0.42 (0.18–0.99)	0.0497	0.523 (0.19–1.47)	0.2175		
Waves	2–3-4 vs. 5 +	0.33 (0.14–0.80)	0.0149	0.232 (0.06–0.88)	**0.0316**	0.26 (0.10–0.67)	**0.0061**
Vaccination	Yes/partly vs. no/not known	0.82 (0.58–1.16)	0.6159	1.259 (0.31–5.07)	0.7455		
Anti-COVID-19 treatment	No vs. yes	2.56 (0.92–8.34)	0.0892	1.729 (0.52–5.72)	0.3690		
Perioperative status	No vs. yes	0.36 (0.13–0.99)	0.0481	0.409 (0.13–1.33)	0.1366	0.340 (0.11–1.01)	0.0527
Hospitalization	Days	0.99 (0.96–1.02)	0.4685	0.988 (0.96–1.02)	0.4671		
Primary tumor	Solitary vs. disseminated (multiple/metastatic/hematologic)	1.58 (0.68–3.69)	0.108	0.959 (0.35–2.60)	0.9351		
Under chemotherapy	No vs. yes	4.09 (1.05–27.18)	0.074	5.063 (0.88–29.1)	0.0689	5.40 (1.26–38.58)	**0.0440**
c) COVID-19 with vs. without pneumonia		Univariable		Multivariable I		Multivariable II (backward selection)	
		OR (95% CI)	*p*	OR (95% CI)	*p*		
Age	Years	1.01 (0.97–1.05)	0.697	1.017 (0.97–1.06)	0.4657		
Sex	Male vs. female	0.46 (0.19–1.07)	0.0721	0.599 (0.22–1.62)	0.3116		
Waves	2–3-4 vs. 5 +	0.36 (0.15–0.86)	0.0225	0.238 (0.06–0.90)	**0.0338**	0.30 (0.12–0.76)	**0.0123**
Vaccination	Yes/partly vs. no/not known	0.856 (0.36–2.08)	0.726	1.568 (0.40–6.23)	0.5228		
Anti-COVID-19 treatment	No vs. yes	2.71 (0.98–8.82)	0.0711	2.004 (0.62–6.47)	0.2452		
Perioperative status	No vs. yes	0.38 (0.13–1.05)	0.062	0.448 (0.14–1.41)	0.1695	0.369 (0.12–1.07)	0.0669
Hospitalization	Days	0.99 (0.97–1.02)	0.6112	0.993 (0.96–1.03)	0.6810		
Primary tumor	Solitary vs. disseminated (multiple/metastatic/hematologic)	1.458 (0.63–3.38)	0.378	0.951 (0.36–2.54)	0.9204		
Under chemotherapy	No vs. yes	2.53 (0.75–11.68)	0.171	2.705 (0.60–2.21)	0.1957	3.067 (0.83–15.17)	0.1196

Univariable and multivariable analyses of which clinical factors significantly influence whether SARS-CoV-2 infection played a direct role in the fatal sequence (a), or any role (direct or contributory) in the fatal sequence (b), or whether pneumonia developed as a complication of SARS-CoV-2 infection (viral pneumonia with or without bacterial overinfection (c)

*OR* odds ratio, *CI* confidence interval

### Population-based mortality outcomes for cancer patients infected with SARS-CoV-2 virus

After quality control and cleaning process, data from 510,149 SARS-CoV-2-infected individuals including 13,693 breast cancer cases, and 899,597 SARS-CoV-2-infected individuals including 10,760 colorectal cancer cases, were available for statistical analysis of COVID-19-related breast and colorectal cancer mortality. The Nagelkerke R Square value assessing the goodness of fit of the logistic regression model was high (breast cancer = 0.613, CRC = 0.612) indicating a strong relationship between the predictors and the outcome for both tumor types. Among SARS-CoV-2-infected patients, women with breast cancer had significantly lower odds of death (OR = 0.817) than the non-breast cancer population. In contrast, the mortality rate of SARS-CoV-2-infected patients with colorectal cancer was significantly higher (OR = 1.254) than that of infected patients without CRC (Supplementary Table 1 and 2.).

## Discussion

This is the first large-scale autopsy-based study analyzing the relationship between the SARS-CoV-2 infection and the cancer-related death.

The studies already published in this field are mainly based on clinical and cancer-registry data which were not confirmed by autopsy findings [17–22]. Accordingly, our findings and those from other approaches are not directly comparable and may be somewhat contradictory. A fundamental finding from the clinical studies is that the COVID-related death rate is generally higher among the cancer patients than in the age-matched general population [17–19, 22]. Nevertheless, a nuanced examination of the published data is warranted. This entails a more detailed analysis, considering factors such as the criteria defining a cancer patient, the specific pandemic waves under consideration, the tissue or organ origin of the cancer, and the age groups being investigated.

In a Canadian study, Hosseini-Moghaddam and colleagues observed an increased risk of SARS-CoV-2 infection, hospitalization, and mortality for individuals with hematologic malignancies compared with the general population, while solid tumor patients had a lower risk of SARS-CoV-2 infection but an increased risk of hospitalization and mortality [19]. However, in a US study investigating patients diagnosed with COVID-19, Chavez-MacGregor and colleagues found that cancer patients who had not undergone recent treatment (these are likely to be patients with cured or currently inactive disease) had similar or better outcomes than non-cancer patients [20]. In contrast, a higher risk of hospitalization, intensive care unit stay, and death was observed in patients who had recently received cancer treatment. Specifically, recent chemotherapy and chemoimmunotherapy were associated with increased mortality of SARS-CoV-2-infected patients. This is supported by our own results, as COVID-19 is more likely to be part of the chain leading to death (underlying disease-complications-direct/immediate cause of death) in patients under active anticancer therapy.

Moreover, as we had access to population-level Hungarian data for two solid tumor types, we investigated mortality among patients suffering from these two cancer types and infected with SARS-CoV-2. Interestingly, among infected women, breast cancer patients had significantly lower odds of death than the non-breast cancer subpopulation. In contrast, the mortality rate of SARS-CoV-2-infected patients with colorectal cancer was significantly higher than that of infected patients without CRC. Therefore, no general conclusions can be drawn regarding the mortality rate of SARS-CoV-2-infected cancer patients from our population-based data. However, this interesting result highlights that differences in the incidence of different tumor types can influence the overall association between COVID-19 and mortality in cancer patients and that our autopsy data may not necessarily contradict the population-based mortality results. Compared to individuals with solitary solid tumors, patients with disseminated disease (metastatic solid tumors, hematologic malignancies) required mechanical ventilation more often and had worse mortality rates [20, 21]. This was in line with our results, as there was a significant difference in the distribution of malignant disease types between SARS-CoV-2-positive and -negative cancer patients (Table 2), with a predominance of hematologic malignancies among SARS-CoV-2-positive cancer patients. On the other hand, we found no significant association between the tumor dissemination status (solitary solid primary tumor versus disseminated disease, including multiple solid primary tumors, metastatic solid tumor, and hematologic malignancies) and whether COVID-19 contributed to patient death, as shown in Table 3.

Bernard et al., analyzing in-hospital mortality rates for subgroups of COVID-19 patients, found that mortality rates for both non-cancer patients and patients with non-metastatic solid cancer, metastatic solid cancer, and hematologic cancer had increased with age [21]. In contrast, no age-related differences were observed in our autopsy cohort (Table 1, Table 2, Supplementary Fig. 1). However, this can be explained both by the much smaller cohort size and by the autopsy nature of our cohort being different from the population data.

The significance of the differences between individual waves is highlighted by a US study by Potter and colleagues analyzed non-cancer and cancer patients who died from COVID-19 during the wild-type (December 2020–February 2021), Delta (July 2021–November 2021), and winter Omicron (December 2021–February 2022) pandemic waves. The results demonstrated that among SARS-CoV-2-infected patients, cancer mortality peaked in a different wave (winter Omicron period) than non-cancer (wild-type period), while strong age-dependent differences were found in the mortality of infected patients with and without cancer in the Delta wave. Differences in mortality by tumor type were also found between the pandemic waves, most notably for patients with lymphoma [23].

Although we did not find such wave-dependent variations in our study cohort, our results also strongly support that differences exist among the individual pandemic waves, of which the second wave was dominated by the wild-type SARS-CoV-2 variant, the third wave by the Alpha, the fourth wave by the Delta, while the 5 + waves were dominated by the Omicron variants in Hungary. We found that both cancer and non-cancer patients were significantly more likely to develop pneumonia in the second wave compared to the 5 + waves and the incidence of pneumonia was significantly higher in non-cancer than cancer patients in the earlier waves, but not in waves 4 and 5 + . Moreover, bacterial overinfection of viral pneumonia among both cancer and non-cancer patients showed a similar increasing trend towards later waves. In the second and third waves, it was significantly more common in cancer patients than in non-cancer patients, and in both groups of patients in waves 5 + compared to the second wave, COVID-19 was not related to the sequence leading to death. The above findings are supported by the results of multivariable analyses showing that earlier than 5 + pandemic waves are independently associated with a higher incidence of pneumonia among SARS-CoV-2-infected cancer patients. Differences between pandemic waves may be explained by differences in the pathogenicity of SARS-CoV-2 variants and the evolving immunological status of the population, which has been influenced by the increasing number of individuals with immunity acquired through vaccination against the virus or recovery from the disease as time has progressed. Our results, furthermore, show that the course of the COVID-19 disease was less severe in the cancer than in the non-cancer patients. SARS-CoV-2 infection without COVID-19 disease was more frequently seen in cancer patients and both viral and bacterial pneumonia were significantly less frequent. In the background of this, we hypothesize the role of altered immune status of the cancer patients. Especially in the earlier waves, the cytokine storm played an important role in the pathogenesis of the COVID-19 disease, including the development of lung alterations, where an extensive immune response and inflammation frequently led to diffuse alveolar damage (DAD) and adult-type acute respiratory distress syndrome (ARDS) [24]. The pathogenetic mechanism of COVID-19 and the mortality data are contradictory, as the excessive inflammatory response characterized by the cytokine storm contributes to COVID-19-induced mortality, while mortality is lower in patients with robust immunity (i.e. young and female patients). On the other hand, population data suggest that impaired immunity, where the conditions for the development of the cytokine storm are less given, is associated with higher mortality (e.g. in immunocompromised and elderly patients) [25]. Based on our results, the latter category also includes the cancer patients, where although the medically detectable symptoms of COVID-19 are milder, the overall mortality is still higher. This discrepancy is reflected also in data such as the fact that in the second wave, the proportion of cancer cases among infected patients who died was higher, while the risk of developing pneumonia was lower, and COVID-19 was not more frequently found as a direct cause of death in cancer patients in wave 2.

In this context, our data also confirm that COVID-19 was less likely to play a direct role in the deaths of cancer patients, more often occurring as a contributory disease or (as a higher proportion of cancer patients had SARS-CoV-2 infection without COVID-19 disease) coexisting morbidity. In our view, this does not really contradict the higher mortality rates associated with COVID-19 in cancer patients observed from clinical studies [17–19, 22], as even a mild additional disease in cancer patients can throw the body out of balance and set off a chain of events leading to death. Therefore, COVID-19 can often be considered only as a contributory disease or even less important coexisting morbidity to the underlying malignant cause of death.

Chavez-MacGregor and colleagues found a higher risk of death in patients who had recently received cancer treatment [20]. Our study supports this, as recent anticancer treatment was the only positive independent predictor of COVID-19 being directly involved in the chain leading to death.

Furthermore, we found an independent negative association between the perioperative status of patients and the direct involvement of COVID-19 in the fatal sequence, suggesting that the underlying malignancy and consequent surgery are more important factors leading to death in these patients than viral disease.

Another interesting finding from the analysis of the autopsy data was that SARS-CoV-2 positivity was more frequent among non-metastatic than metastatic cancer cases. This can be related to the observation of Hosseini-Moghaddam and colleagues that solid tumor patients had a lower risk of SARS-CoV-2 infection [19]. One of the factors behind the lower incidence of SARS-CoV-2 infection in patients with solid tumors and especially in patients with metastatic cancer, and the milder course of COVID-19, may be the immunological status influenced by SARS-CoV-2 infection. However, other studies also did not confer metastatic disease as a significant risk of severe COVID-19 disease [4].

This study, like most studies usually, has certain limitations. One of the main limitations is that although we know which patients were COVID-19 positive at the time of death, we do not know who had previously experienced the disease without laboratory confirmation of the SARS-CoV-2 positivity. A previous study found that SARS-CoV-2-positive patients with a history of cancer in the anamnestic data had a higher rate of serious events than the non-cancer population. Our study included a total of 57 patients with a history of cancer who were treated surgically and survived without complications. Among these patients, a total of 7 patients were SARS-CoV-2 positive, so with this number of cases, we could not handle this subgroup during the statistical analyses separately [12]. The retrospective nature of the study is also a limiting factor. As well as, we have not been able to take into account that vaccination status and pandemic waves are not independent of each other (more vaccinated people appear in later waves). Another major limitation is that it is not based on multicentre, large-scale data collection, but on autopsy results from a single tertiary COVID-19 centre, where the incidence of different cancer types may not be the same as the population average. Due to the diversity of patients’ malignant disease, it was not possible to assess their advancement in progession or status in a uniform way. In our study, we did not have the opportunity to examine the first wave, because during this period, SARS-CoV-2-positive deceased patients were only autopsied in particular settings. Although it is widely known that lesions caused by SARS-CoV-2 infection can occur in a variety of extrapulmonary localizations, the present study is limited to the analysis of pulmonary manifestations [26, 27].

To conclude our autopsy study, the course of COVID-19 in cancer patients was much more balanced during the pandemic than in non-cancer patients, who more often had severe, fatal COVID-19 in the early disease waves. This may be partly due to the relative immunosuppressed status of cancer patients, and to the fact that even early/mild viral infection can more easily upset the balance of their condition, leading to death from their underlying cancer or its other complications.

## Supplementary Information

Below is the link to the electronic supplementary material.

Supplementary file1 (PDF 199 KB)

## References

[1] Wang H, Paulson KR, Pease SA, et al. Estimating excess mortality due to the COVID-19 pandemic: a systematic analysis of COVID-19-related mortality, 2020–21. The Lancet. 2022;399:1513–36. 10.1016/S0140-6736(21)02796-3. 10.1016/S0140-6736(21)02796-3 PMC8912932 35279232

[2] Horvath J, Krisztina K, Katalin K, et al. A COVID-19 világjárvány első két éve Magyarországon. The first two years of the COVID-19 pandemic in Hungary. Népegészségügy. 2022;99:6–17.

[3] Sica A, Colombo MP, Trama A, et al. Immunometabolic status of COVID-19 cancer patients. Physiol Rev. 2020;100:1839–50. 10.1152/physrev.00018.2020. 32721181 10.1152/physrev.00018.2020 PMC7839651

[4] Robilotti EV, Babady NE, Mead PA, et al. Determinants of severity in cancer patients with COVID-19 illness. Nat Med. 2020;26:1218–23. 10.1038/s41591-020-0979-0. 32581323 10.1038/s41591-020-0979-0 PMC7785283

[5] Onder G, Rezza G, Brusaferro S. Case-fatality rate and characteristics of patients dying in relation to COVID-19 in Italy. JAMA. 2020;323:1775–6. 10.1001/jama.2020.4683. 32203977 10.1001/jama.2020.4683

[6] Li Y-S, Ren H-C, Cao J-H. Correlation of SARS-CoV-2 to cancer: carcinogenic or anticancer? (Review). Int J Oncol. 2022;60:42. 10.3892/ijo.2022.5332. 35234272 10.3892/ijo.2022.5332 PMC8923649

[7] Hiam-Galvez KJ, Allen BM, Spitzer MH. Systemic immunity in cancer. Nat Rev Cancer. 2021;21:345–59. 10.1038/s41568-021-00347-z. 33837297 10.1038/s41568-021-00347-z PMC8034277

[8] Grivas P, Khaki AR, Wise-Draper TM, et al. Association of clinical factors and recent anticancer therapy with COVID-19 severity among patients with cancer: a report from the COVID-19 and Cancer Consortium. Ann Oncol. 2021;32:787–800. 10.1016/j.annonc.2021.02.024. 33746047 10.1016/j.annonc.2021.02.024 PMC7972830

[9] Zsichla L, Müller V. Risk factors of severe COVID-19: a review of host, viral and environmental factors. Viruses. 2023;15:175. 10.3390/v15010175. 36680215 10.3390/v15010175 PMC9863423

[10] Yu J, Ouyang W, Chua MLK, Xie C. SARS-CoV-2 transmission in patients with cancer at a tertiary care hospital in Wuhan, China. JAMA Oncol. 2020;6:1108–10. 10.1001/jamaoncol.2020.0980. 32211820 10.1001/jamaoncol.2020.0980 PMC7097836

[11] Wang D, Hu B, Hu C, et al. Clinical characteristics of 138 hospitalized patients with 2019 novel coronavirus-infected pneumonia in Wuhan, China. JAMA. 2020;323:1061–9. 10.1001/jama.2020.1585. 32031570 10.1001/jama.2020.1585 PMC7042881

[12] Liang W, Guan W, Chen R, et al. Cancer patients in SARS-CoV-2 infection: a nationwide analysis in China. Lancet Oncol. 2020;21:335–7. 10.1016/S1470-2045(20)30096-6. 32066541 10.1016/S1470-2045(20)30096-6 PMC7159000

[13] Bange EM, Han NA, Wileyto P, et al. CD8+ T cells contribute to survival in patients with COVID-19 and hematologic cancer. Nat Med. 2021;27:1280–9. 10.1038/s41591-021-01386-7. 34017137 10.1038/s41591-021-01386-7 PMC8291091

[14] Dai M, Liu D, Liu M, et al. Patients with cancer appear more vulnerable to SARS-CoV-2: a multicenter study during the COVID-19 outbreak. Cancer Discov. 2020;10:783–91. 10.1158/2159-8290.CD-20-0422. 32345594 10.1158/2159-8290.CD-20-0422 PMC7309152

[15] Róka E, Déri D, Khayer B, et al. SARS-CoV-2 variant detection from wastewater: rapid spread of B.1.1.7 lineage in Hungary. J Water Health. 2022;20:277–86. 10.2166/wh.2022.179. 36366986 10.2166/wh.2022.179

[16] Kiss Z, Wittmann I, Polivka L, et al. Nationwide effectiveness of first and second SARS-CoV2 booster vaccines during the delta and omicron pandemic waves in Hungary (HUN-VE 2 study). Front Immunol. 2022;13:905585. 10.3389/fimmu.2022.905585. 35812442 10.3389/fimmu.2022.905585 PMC9260843

[17] Nadkarni AR, Vijayakumaran SC, Gupta S, Divatia JV. Mortality in cancer patients with COVID-19 who are admitted to an ICU or who have severe COVID-19: a systematic review and meta-analysis. JCO Glob Oncol. 2021;7:1286–305. 10.1200/GO.21.00072. 34406802 10.1200/GO.21.00072 PMC8457815

[18] Giannakoulis VG, Papoutsi E, Siempos II. Effect of cancer on clinical outcomes of patients with COVID-19: a meta-analysis of patient data. JCO Glob Oncol. 2020;6:799–808. 10.1200/GO.20.00225. 32511066 10.1200/GO.20.00225 PMC7328119

[19] Hosseini-Moghaddam SM, Shepherd FA, Swayze S, et al. SARS-CoV-2 infection, hospitalization, and mortality in adults with and without cancer. JAMA Netw Open. 2023;6:e2331617. 10.1001/jamanetworkopen.2023.31617. 37651139 10.1001/jamanetworkopen.2023.31617 PMC10472189

[20] Chavez-MacGregor M, Lei X, Zhao H, et al. Evaluation of COVID-19 mortality and adverse outcomes in US patients with or without cancer. JAMA Oncol. 2022;8:69–78. 10.1001/jamaoncol.2021.5148. 34709356 10.1001/jamaoncol.2021.5148 PMC8554684

[21] Bernard A, Cottenet J, Bonniaud P, et al. Comparison of cancer patients to non-cancer patients among COVID-19 inpatients at a national level. Cancers. 2021;13:1436. 10.3390/cancers13061436. 33801131 10.3390/cancers13061436 PMC8004216

[22] Abuhelwa Z, Alsughayer A, Abuhelwa AY, et al. In-hospital mortality and morbidity in cancer patients with COVID-19: a nationwide analysis from the United States. Cancers. 2022;15:222. 10.3390/cancers15010222. 36612218 10.3390/cancers15010222 PMC9818639

[23] Potter AL, Vaddaraju V, Venkateswaran S, et al. Deaths due to COVID-19 in patients with cancer during different waves of the pandemic in the US. JAMA Oncol. 2023;9:1417–22. 10.1001/jamaoncol.2023.3066. 37651113 10.1001/jamaoncol.2023.3066 PMC10472259

[24] de la Rica R, Borges M, Gonzalez-Freire M. COVID-19: In the eye of the cytokine storm. Front Immunol. 2020;11:558898. 10.3389/fimmu.2020.558898. 33072097 10.3389/fimmu.2020.558898 PMC7541915

[25] Alcock J, Masters A. Cytokine storms, evolution and COVID-19. Evol Med Public Health. 2021;9:83–92. 10.1093/emph/eoab005. 34552755 10.1093/emph/eoab005 PMC7928963

[26] Danics K, Pesti A, Törő K, et al. A COVID-19-association-dependent categorization of death causes in 100 autopsy cases. GeroScience. 2021;43:2265–87. 10.1007/s11357-021-00451-w. 34510338 10.1007/s11357-021-00451-w PMC8435112

[27] Pesti A, Danics K, Glasz T, et al. Liver alterations and detection of SARS-CoV-2 RNA and proteins in COVID-19 autopsies. GeroScience. 2023;45:1015–31. 10.1007/s11357-022-00700-6. 36527584 10.1007/s11357-022-00700-6 PMC9759055

