# Supplementary material for: Autopsy findings in cancer patients infected with SARS-CoV-2 show a milder presentation of COVID-19 compared to non-cancer patients
Source: GeroScience. 2024 Apr 30;46(6):6101–14. doi: 10.1007/s11357-024-01163-7 (PMC11493920; doi:10.1007/s11357-024-01163-7)
Supplement: Supplementary file 1 — Supplementary file1 (PDF 199 KB) [file 11357_2024_1163_MOESM1_ESM.pdf]

Supplementary Material
for

Autopsy findings in cancer patients infected with SARS-CoV-2 show a milder presentation of COVID-19 compared to non-cancer patients

Éva Kocsmár*#[1], Ildikó Kocsmár*[1,2], Flóra Elamin[1], Laura Pápai[1], Ákos Jakab[1], Tibor Várkonyi[1] , Tibor Glasz[1], Gergely Rácz[3], Adrián Pesti[1], Krisztina Danics[1], András Kiss[1], Gergely Röst[4,5], Éva Belicza[6], Zsuzsa Schaff[1], and Gábor Lotz#[1]

[1]Department of Pathology, Forensic and Insurance Medicine, Semmelweis University

[2]Department of Urology, Semmelweis University, Budapest, Hungary

[3]Department of Pathology and Experimental Cancer Research, Semmelweis University, Budapest, Hungary

[4]National Laboratory for Health Security, University of Szeged, Szeged, Hungary

[5]Hungarian Centre of Excellence for Molecular Medicine (HCEMM), Szeged, Hungary

[6]Health Services Management Training Centre, Faculty of Health and Public Administration, Semmelweis University, Budapest, Hungary

*These authors contributed equally.

#Correspondence to:

Gábor Lotz MD PhD e-mail: lotz.gabor@med.semmelweis-univ.hu

Éva Kocsmár MD PhD e-mail: kocsmar.eva@semmelweis.hu

Supplementary Material
for the Methods

Database query details of the population-based mortality data and results

Colorectal cancer (CRC) patients were identified based on the ICD-10 codes C17-C20 (CRC ICD). In more detail, CRC patients were included in the study if they had a CRC ICD code in an inpatient clinical care setting and one of the following: a CRC specific morphology code (SNOMED) or surgery code (ICPM - International classification of Procedures in Medicine) OR had a CRC ICD code in an inpatient or outpatient clinical care setting and also had a specific radiation or chemotherapy ICPM code OR the patient was treated under a CRC ICD code at an inpatient clinical oncology or inpatient/outpatient radiotherapy site, or diagnosed with a CRC ICD code at an outpatient pathology service.

Breast cancer patients were identified based on the ICD-10 code C50. In more detail, breast cancer patients were included in the study if they had the C50 ICD code in an inpatient clinical care setting and one of the following: a C50 specific morphology code (SNOMED) or surgery code (ICPM - International classification of Procedures in Medicine) OR had the C50 ICD code in an inpatient or outpatient clinical care setting and also had a specific radiation or chemotherapy ICPM code OR the patient was treated under the C50 ICD code at an inpatient clinical oncology or inpatient/outpatient radiotherapy site, or diagnosed with the C50 ICD code at an outpatient pathology service.

The comorbidities studied and their ICD codes (obtained either from inpatient or outpatient care included in the patient's medical history from 2010 until the patient's death or the end of the study period): Diabetes (E10-E14), dementia (F00-F07), depression (F31-F33, F41, F9200), neurological disorders (G541, G543, G544, G55), hypertension (I10), hypertensive heart disease (I11), other malignancies [for colorectal cancers: ICD beginning with C except basal cell carcinoma (C44), metastasis (C78-C79) and C17-C20 ; for breast cancer: ICD beginning with letter C, except basal cell carcinoma (C44), metastasis (C78-C79) and C50], myocardial infarction (I21-I23), malignant tumor metastasis (C78-C79), renal disease (N188, N189), liver disease (K70-K73, K740-K742, K746-K749, K752,K753,K758, K759, K760, K766, K767, K769), chronic obsructive pulmonary disease - COPD (J99, J40-J42, J431-J439, J44, J47, J96, J980-J984, J988-J989), stroke (I63-I64), vascular diseases (I70-I78, I790, I792-I799), heart failure (I50), other ischaemic heart diseases (I20, I24, I25), cerebrovascular diseases (I65-I69), rheumatic heart and other diseases I00-I09. Reference: shown in the table, otherwise the first category of ordinal variables and the "no" category of binary (yes/no) variables.

The odds of death increase with age but decrease with the number of SARS-CoV-2 vaccinations and infections. Breast cancer improved survival by about 17%. CRC increases the odds of death by about 25% overall, but women with CRC have a significantly better chance of survival than men.

Supplementary Figure

Differences by age

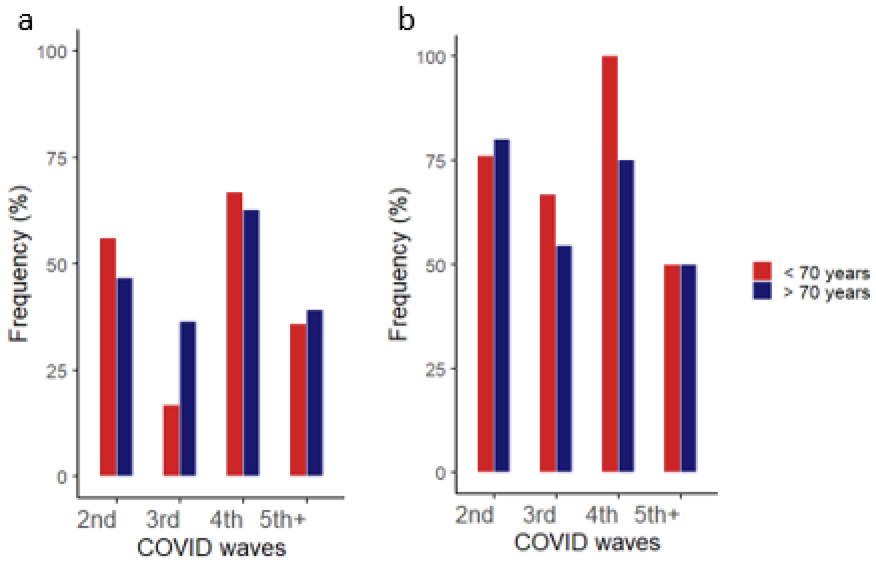

Supplementary Figure 1 - Analysis of age differences by COVID-19 waves

No significant differences were found between patients aged 70 years or younger and those aged 70 years or older by wave, either in terms of the role of COVID-19 disease in the fatal sequence leading to death (paired Wilcoxon test, p=1, Supplementary Figure a), or in the proportion of patients developing pneumonia (p=0.4227, Supplementary Figure b).

Supplementary Tables

Parameter		n	B	SE	Wald	df	P	OR
**Age**	40-59 years (ref)	331783			14088.759	2	0.000	
	60-79 years	136968	2	0.024	5942.151	1	0.000	6.297
	80 years ≤	41398	3	0.027	13846.391	1	0.000	25.285
**Breast cancer**	**present**	13693	**0**	**0.038**	**23.652**	**1**	**0.000**	**0.831**
**Number of vaccine doses**	0 (ref)	149202			21809.863	5	0.000	
	1	25195	-1	0.030	642.166	1	0.000	0.472
	2	241769	-2	0.019	13944.748	1	0.000	0.109
	3	93724	-3	0.028	13825.429	1	0.000	0.038
	4	254	-3	0.416	61.657	1	0.000	0.038
	5	5	-19	15791.781	0.000	1	0.999	0.000
**Number of SARS-CoV-2 infections**	1 (ref)	490778			176.050	2	0.000	
	2	19215	-1	0.057	166.242	1	0.000	0.481
	3	156	-2	0.481	10.086	1	0.001	0.217
**Medical history of comorbidities**	Diabetes mellitus	87638	0	0.017	205.402	1	0.000	1.274
	Dementia	41548	1	0.020	3437.980	1	0.000	3.151
	Depression	122739	0	0.018	361.050	1	0.000	0.715
	Neurological disorders	3663	0	0.077	4.191	1	0.041	0.855
	Hypertension	298206	0	0.027	282.644	1	0.000	1.569
	Hypertensive heart disease	24412	0	0.023	24.321	1	0.000	1.120
	Ischaemic heart disease	14093	0	0.030	53.453	1	0.000	0.801
	Myocardial infarction	9178	0	0.035	0.058	1	0.810	0.992
	Heart failure	48216	1	0.018	4999.840	1	0.000	3.582
	Rheumatic and other heart diseases	128320	0	0.018	112.512	1	0.000	0.823
	Cerebrovascular diseases	73591	0	0.019	9.847	1	0.002	0.942
	Vascular diseases	114472	0	0.017	482.180	1	0.000	1.465
	Stroke	42672	0	0.020	262.244	1	0.000	1.390
	COPD	113158	1	0.016	6047.822	1	0.000	3.482
	Liver disease	54440	0	0.021	3.181	1	0.074	0.962
	Renal disease	31507	1	0.020	919.967	1	0.000	1.833
	Malignant tumor metastasis	8168	2	0.037	2245.927	1	0.000	5.725
	Other malignant diseases*	42304	1	0.021	680.174	1	0.000	1.751
	Constant		-5	0.026	30560.647	1	0.000	0.010

Supplementary Table 1 - Population-based mortality outcomes for breast cancer patients infected with SARS-CoV-2 virus by analyzing data from 510,149 individuals

Nagelkerke R Square value: 0.613; Abbreviations: B - coefficient for the constant, CI - confidence interval, df - degrees of freedom, n - number of individuals, OR - odds ratio, P - p value, ref - reference, SE - standard error, Wald - Wald value.

* Excluding basal cell carcinoma, malignant tumor metastasis and breast cancer.

Parameter		n	B	SE	Wald	df	P	OR
**Sex**	female<br>(ref: male)	510149<br>(389448)	-1	0,012	2183,738	1	0,000	0,577
**Age**	40-59 years (ref)	594729			24508,220	2	0,000	
	60-79 years	247310	2	0,015	12092,239	1	0,000	5,457
	80 years ≤	57 558	3	0,020	24399,969	1	0,000	22,244
**Colorectal cancer**	present	10760	0	0,033	48,322	1	0,000	1,254
**Number of vaccine doses**	0 (ref)	268634			42848,892	5	0,000	
	1	46466	-1	0,021	1272,830	1	0,000	0,468
	2	414070	-2	0,014	27512,034	1	0,000	0,098
	3	169946	-3	0,021	27374,076	1	0,000	0,032
	4	470	-3	0,329	109,084	1	0,000	0,032
	5	11	-19	11195,853	0,000	1	0,999	0,000
**Number of SARS-CoV-2 infections**	1 (ref)	869039			400,795	2	0,000	
	2	30313	-1	0,044	387,396	1	0,000	0,422
	3	245	-1	0,358	13,867	1	0,000	0,264
**Medical history of comorbidities**	Diabetes mellitus	161630	0	0,012	433,991	1	0,000	1,297
	Dementia	63579	1	0,015	5463,251	1	0,000	3,120
	Depression	175210	0	0,014	469,308	1	0,000	0,740
	Neurological disorders	5722	0	0,060	3,546	1	0,060	0,893
	Hypertension	524390	0	0,018	458,758	1	0,000	1,466
	Hypertensive heart disease	45399	0	0,017	74,884	1	0,000	1,159
	Ischaemic heart disease	23111	0	0,024	72,235	1	0,000	0,819
	Myocardial infarction	23215	0	0,023	7,631	1	0,006	0,937
	Heart failure	8797	1	0,013	9924,921	1	0,000	3,818
	Other heart diseases	226234	0	0,014	328,349	1	0,000	0,781
	Cerebrovascular diseases	121276	0	0,015	16,243	1	0,000	0,943
	Vascular diseases	189436	0	0,013	816,738	1	0,000	1,449
	Stroke	74115	0	0,016	629,835	1	0,000	1,476
	COPD	203795	1	0,012	15316,725	1	0,000	4,324
	Liver disease	106956	0	0,015	26,015	1	0,000	1,079
	Renal disease	52672	1	0,015	1713,534	1	0,000	1,892
	Malignant tumor metastasis	14984	2	0,027	3889,496	1	0,000	5,398
	Other malignant diseases*	83722	0	0,016	913,271	1	0,000	1,599
	Constant		-4	0,017	52537,084	1	0,000	0,019

Supplementary Table 2 - Population-based mortality outcomes for colorectal cancer patients infected with SARS-CoV-2 virus by analyzing data from 899,597 individuals

The Nagelkerke R Square value: 0.612; Abbreviations: B - coefficient for the constant, CI - confidence interval, df - degrees of freedom, n - number of individuals, OR - odds ratio, P - p value, ref - reference, SE - standard error, Wald - Wald value.

* Excluding basal cell carcinoma, malignant tumor metastasis and colorectal cancer.
